# Dark and Photoinduced Cytotoxic Activity of the New Chlorophyll-a Derivatives with Oligoethylene Glycol Substituents on the Periphery of Their Macrocycles

**DOI:** 10.3390/ijms18010103

**Published:** 2017-01-05

**Authors:** Yana I. Pylina, Dmitry M. Shadrin, Oksana G. Shevchenko, Olga M. Startseva, Igor O. Velegzhaninov, Dmitry V. Belykh, Ilya O. Velegzhaninov

**Affiliations:** 1 Institute of Biology of Komi Science Centre of Ural Branch of RAS, Syktyvkar 167982, Russia; yanapylina@yandex.ru (Y.I.P.); shdimas@yandex.ru (D.M.S.); microtus69@mail.ru (O.G.S.); 2 Institute of Chemistry of Komi Science Centre of Ural Branch of RAS, Syktyvkar 167982, Russia; om_smirnova@mail.ru (O.M.S.); belykh-dv@mail.ru (D.V.B.); 3 Institute of Geology of Komi Science Centre of Ural Branch of RAS, Syktyvkar 167982, Russia; saturn_vio@mail.ru

**Keywords:** photosensitizer, photodynamic therapy, HeLa, photohemolysis, chlorophyll-a derivatives, oligoethylene glycol substituent, cytotoxic activity

## Abstract

In the present work, we investigated the dark and photoinduced cytotoxic activity of the new chlorophyll-a derivatives which contain the substituents of oligoethylene glycol on the periphery of their macrocycles. These compounds were tested using human cell lines to estimate their potential as photosensitizers for photodynamic therapy of cancer. It was shown that all the tested compounds have expressed photoinduced cytotoxic activity in vitro. Detailed study of the biological activity of one of the most perspective compound in this series—pyropheophorbide-a 17-diethylene glycol ester (Compound **21**) was performed. This new compound is characterized by lower dark cytotoxicity and higher photoinduced cytotoxicity than previously described in a similar compound (DH-I-180-3) and clinically used Photolon^TM^. Using fluorescent microscopy, it was shown that Compound **21** quickly penetrates the cells. Analysis of caspase-3 activity indicated an apoptosis induction 40 min after exposure to red light (λ = 660 nm). The induction of DNA damages and apoptosis was shown using Comet assay. The results of expression analysis of the stress-response genes indicate an activation of the genes which control the cell cycle and detoxification of the free radicals after an exposure of HeLa cells to Compound **21** and to red light. High photodynamic activity of this compound and the ability to oxidize biomolecules was demonstrated on nuclear-free mice erythrocytes. In addition, it was shown that Compound **21** is effectively activated with low energy 700 nm light, which can penetrate deep into the tissue. Thus, Compound **21** is a prospective substance for development of the new drugs for photodynamic therapy of cancer.

## 1. Introduction

Chlorophyll-a derivatives are used in medicine as photosensitizers for photodynamic therapy of cancer (PDT) [1,2,3,4,5,6,7]. PDT is considered to be a modern prospective method which is based on accumulation of the photosensitizer in the tumor tissue. After an exposure to the specific wavelength of light, this photosensitizer produces free radicals which damage the structural elements of the tumor tissue [6,8]. An important property of photosensitizers is amphiphilicity which in many cases provides a high antineoplastic activity [9]. Chlorophyll-a derivatives with oligoethylene glycol substituents on the periphery of their macrocycles can be used as these prospective amphiphilic photosensitizers [10,11,12].

In the present work, we show the results of the research of the dark and photoinduced cytotoxic activity of the new chlorophyll-a derivatives with one substituent of oligoethylene glycol [13] (where the number of ethylene glycol units varies from two to six) which is linked to the chlorin macrocycle with an ester bond. We describe a detailed research of biological activity in vitro of pyropheophorbide-a 17-diethylene glycol ester (Compound **21**), the most perspective one of the compounds from the researched group.

## 2. Results and Discussion

### 2.1. The Dark Cytotoxic Activity of Chlorophyll-a Derivatives with Oligoethylene Glycol Substituents

**Figure 1:**
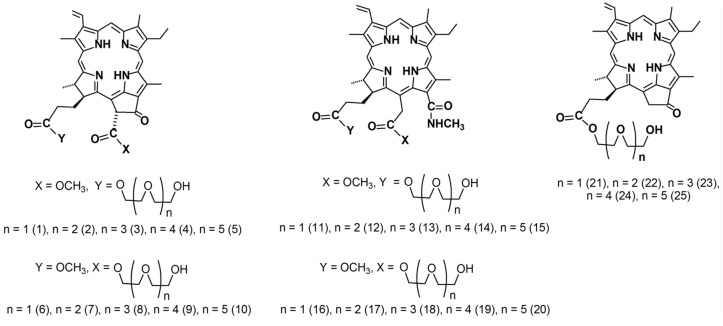
Structures of tested chlorophyll-a derivatives.

Using HeLa cells 25, chlorophyll-a derivatives which contain the substituents of oligoethylene glycol on the periphery of their macrocycles were tested on the presence of dark cytotoxic activity (Figure 1). It should be noted that the impact of oligoethylene glycol introduction on dark cytotoxicity is complex and depends on the structure of the macrocycle and position of the polyester substitute. Incorporation of the same oligoethylene glycol substituent into different chlorophyll-a derivatives can lead to both an increase and decrease of the dark cytotoxicity (Table 1). Elongation of the polyester chain leads to some decrease of the cytotoxicity in the series of methyl pheophorbide a 13(2)-esters (**6**–**10**) and at the same time it leads to an increase in cytotoxic activity of 17-esters of methyl pheophorbide-a (**1**–**5**) and methyl pyropheophorbide-a (**21**–**25**). In case of methylamide derivatives with polyester substituents at the position 15, the derivatives with an even number of links in the polyester chain are more toxic than the derivatives with an odd number of links (**16**–**20**). However, elongation of oligoethylene glycol substituent of methylamide derivatives at the position 17 leads to a decrease of toxicity (**11**–**15**). It is worth noting that all oligoethylene glycols in the concentration of 100 μM were not toxic (Table 1).

Thus, the analysis of dark cytotoxicity of chlorophyll-a derivatives shows that introduction of oligoethylene glycol substituents in some cases can lead to a noticeable increase of dark cytotoxicity. Different influence from the introduction of the same oligoethylene glycol substituent into different chlorophyll-a derivatives indicates that the observed biological activity is caused by action of unhydrolyzed molecules.

### 2.2. Photoinduced Cytotoxic and Cytostatic Activity of Chlorophyll-a Derivatives with Oligoethylene Glycol Substituents

Earlier, Lim and colleagues (2003, 2006) determined both in vitro and in vivo that the Compound **7** is an effective photodynamic antineoplastic agent [10,11]. Later, Park and colleagues (2015) showed that the use of lipid nanoparticles filled with this compound increase their delivery precision and photocytotoxic effect [14]. That is why the tested structural series of compounds can be considered to be a perspective source for the search of new photosensitizers with improved properties for PDT of cancer.

**Figure 2:**
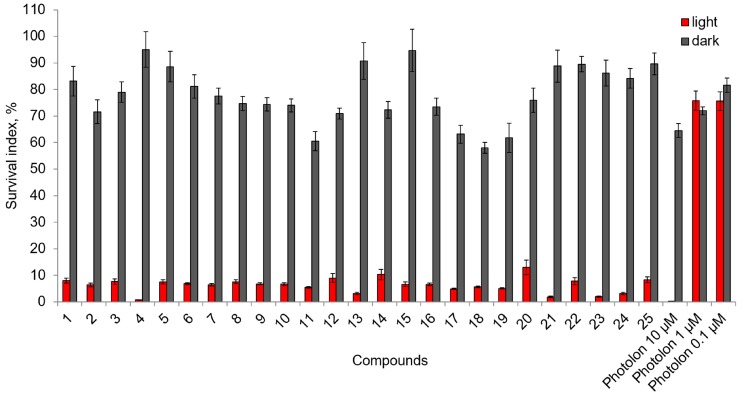
Photoinduced cytotoxic activity of chlorophyll-a derivatives (1 μM) and of Photolon^TM^ (0.1, 1 and 10 μM) activated by 660 nm light (2 h of preincubation then 20 min light exposure followed by incubation in the dark for 70 h).

The results of our experiment on HeLa cells indicated that the majority of the tested chlorophyll-a derivatives with oligoethylene glycol substituents had a photoinduced cytotoxicity at a concentration of 1 μM after activation with light (λ = 660 nm) (Figure 2). In comparison, the famous medical drug Photolon^TM^ becomes photoactive only at a much higher concentration.

Compound **21** is characterized by low dark cytotoxicity (Table 1), while in concentration of 1 μM this compound has a high photoinduced cytotoxic effect. The difference between the dark and photoinduced toxicity of minimum one order of magnitude gives a strong reason to view this derivative as a perspective substance for further research oriented on development of the new drugs for PDT of cancer.

**Figure 3:**
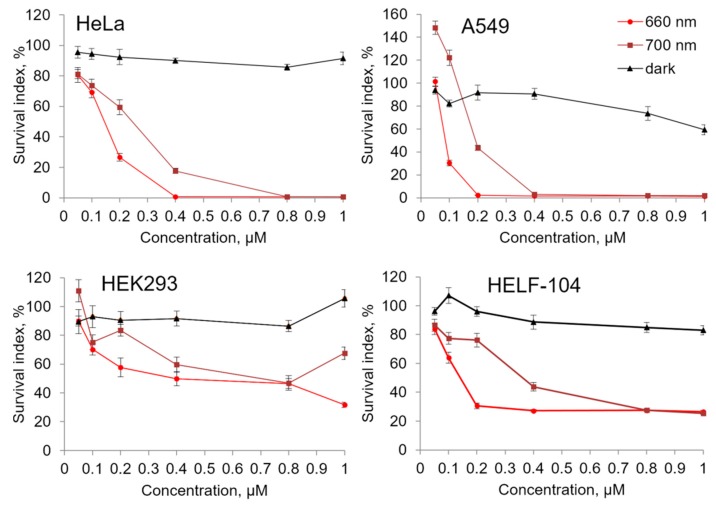
Photoinduced cytotoxic activity of Compound **21** against four cell lines (HeLa, A549, HEK293, and HELF-104). After 2 h of preincubation, the tested compound was photoactivated by 20 min exposure to 660 or 700 nm light followed by incubation in the dark for 70 h.

Because Compound **21** showed lowest dark cytotoxicity among tested photoactive substances, it was studied in detail to analyze the correlation between its concentration and cytotoxic activity (Figure 3). We conducted additional tests to determine the survival of the cancerous cell lines: HeLa, Hek293, A549, and the survival of normal human embryonic lung fibroblasts (HELF-104) in the range of Compound **21** concentration from 0.05 to 1 μM activated with red light (λ = 660 nm) (Figure 3). It was determined that the tested compound showed a rapid increase of photoinduced cytotoxicity in the concentration range 0.1–0.4 μM. Photoinduced cytotoxicity of the tested compound on the cell lines HeLa, A549, and HELF-104 (Table 2) was one order of magnitude higher than that of Photolon^TM^. For the Hek293 cell line, the difference between IC_50_ of these two compounds was less significant. Besides that, while having a high photoinduced toxicity, Compound **21** was much less toxic in the dark than the Compound **7** which was researched earlier [10,11] (Table 1).

It is known that the ability of light to penetrate tissue increases as the wavelength increases and the photon’s energy and ability to activate photosensitizer to generate singlet oxygen decreases. Because of this, an important property of a photosensitizer is its ability to be activated in response to long-wave light exposure. The results which are represented on the in Figure 3 show that Compound **21** is effectively activated with the 700 nm light.

### 2.3. Cytostatic and Cytotoxic Photoinduced Activity of Compound ***21***

**Figure 4:**
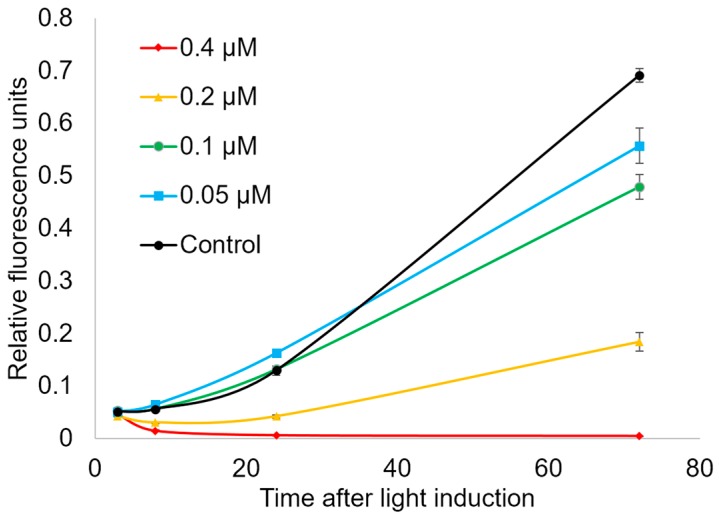
The growth dynamics of HeLa microcultures treated with Compound **21** with photoinduction (20 min, 660 nm). Relative fluorescence units indicate relative amount of fluorescein diacetate hydrolyzed by interaction with intact cell membranes for 40 min. This value is proportional to the number of cells in the well plate.

The analysis of the amount of dynamic of living cells after light exposure in the presence of Compound **21** was performed using FMCA. The results indirectly showed both cytostatic and cytotoxic activity of the photosensitizer. Treatment of HeLa cells with the compound at concentrations 0.05 and 0.1 µM did not reduce the ability of microcultures to hydrolyze fluorescein diacetate 3, 8, and 24 h after photoinduction. However, 70 h after light induction, this parameter has a lower value than in control microcultures. This fact can be explained by cytostatic activity of the compound and by causing of reproductive cell death (Figure 4). It should be noted that 72 h incubation of the cells with Compound **21** in the tested concentrations without photoinduction did not lead to a decreasing of number of living cells.

In order to verify the cytostatic activity of Compound **21**, we analyzed the expression of the genes: *CDKN1A* (*p21*), *CDKN2A* (*p16*), and *CDKN2D* (*p19*), which code for cyclin-dependent kinases of the cell cycle checkpoints (Table 3). An increase in the expression of genes *CDKN1A* and *CDKN2D* happened under the action of photoactivated Compound **21** at a concentration of 0.4 μM. This indicates an induction of cell cycle arrest in some of the cells. The cyclin-dependent kinases’ expression level was not changed after treatment of cells with the same concentration of Compound **21** without light exposure.

### 2.4. Ability of Compound ***21*** to Penetrate the Plasmalemma

We used the autofluorescence of Compound **21** to determine its ability to penetrate the plasmalemma. Analysis of the fluorescence spectrum of the water solution of the compound (0.5 μM) showed a distinct photon emission with the maximum intensity at λ = 660 nm provided that the exciting wavelength was lower than 350 nm with a maximum at 330 nm. The water solution was prepared the same way as for biotests, with a transitional step of dissolving in DMSO.

**Figure 5:**
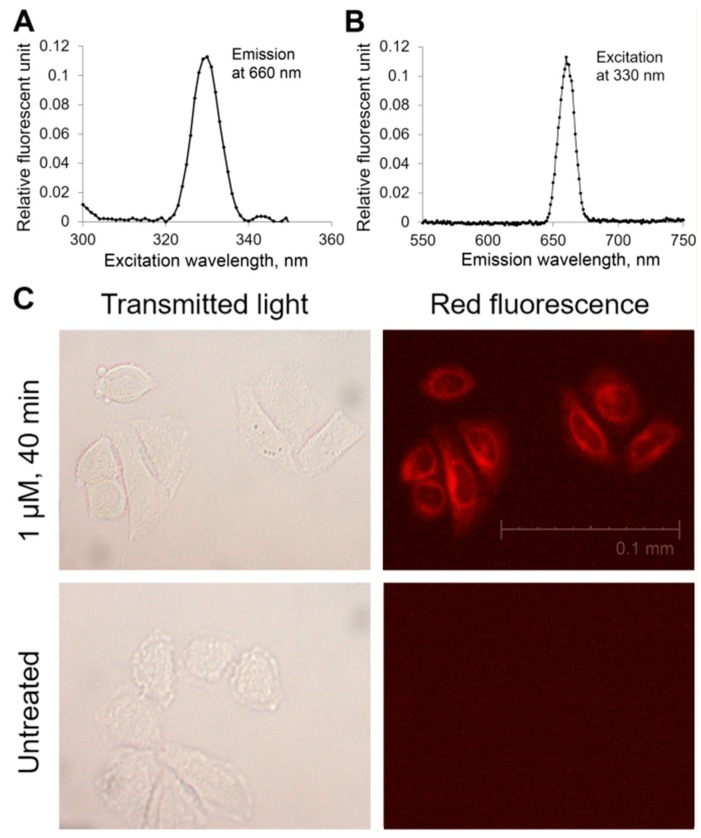
Spectrum of fluorescence excitation (**A**) and emission (**B**) of 0.5 µM Compound **21** solution. Light micrographs (**C**, **left** column) and fluorescent micrographs (**C**, **right** column) of cells after 40 min treatment with Compound **21** (**C**, **upper** row) and without it (**C**, **lower** row). Magnification: 600×.

The use of fluorescent microscopy allowed us to visualize accumulation of the tested Compound **21** in the cell after incubation with it (Figure 5). The cells which were incubated for 40 min in the growth media, containing 1 μM of Compound **21**, and then washed with PBS emit fluorescence in the red spectrum. Fluorescence was not distributed evenly in the cell. The cytoplasm was stained more than the nucleus. The fluorescence was not observed if the cells were incubated in the same growth medium with DMSO but without Compound **21**. This fact allows us to make a conclusion that the compound penetrates into the cells.

### 2.5. Genotoxic and Apoptosis-Induced Activity of Compound ***21***

**Figure 6:**
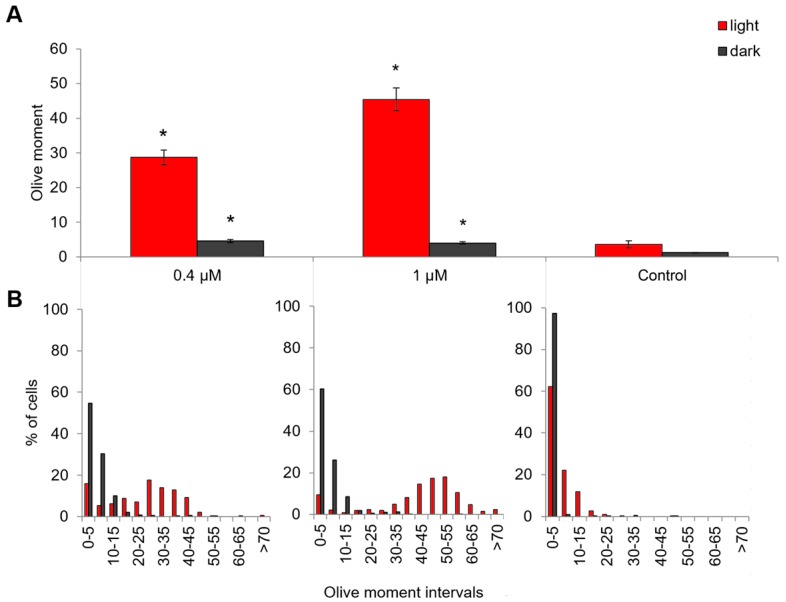
The level of DNA damage in HeLa cells in the dark and after activation with light (λ = 660 nm), estimated with Comet assay (2 h in the dark then 20 min under red light then 6 h in the dark). At the Figure (**A**), there are average values of the six experimental repeats, within each repeat 50–80 cells were analyzed; At the Figure (**B**) the distribution of the number of cells according to Olive moment is shown with the interval being equal to five units in each variant. *—the difference from control (Solvent—DMSO in the dark) is significant at *p* < 0.005.

Using the alkaline version of the Comet assay, we tested the ability of Compound **21** (0.4 and 1 μM) to induce the DNA damage in HeLa cells in the dark and after activation with light (λ = 660 nm) (Figure 6A). It was shown that the photoactivated Compound **21** in the concentrations of 0.4 and 1 μM increased the DNA damage level 6- and 11-fold respectively relative to control (the same compound concentration, but no light). The mean value of the Olive moment increases because of emergence of a separate pool of highly damaged cells after the action of the compound in both concentrations (Figure 6B).

**Figure 7:**
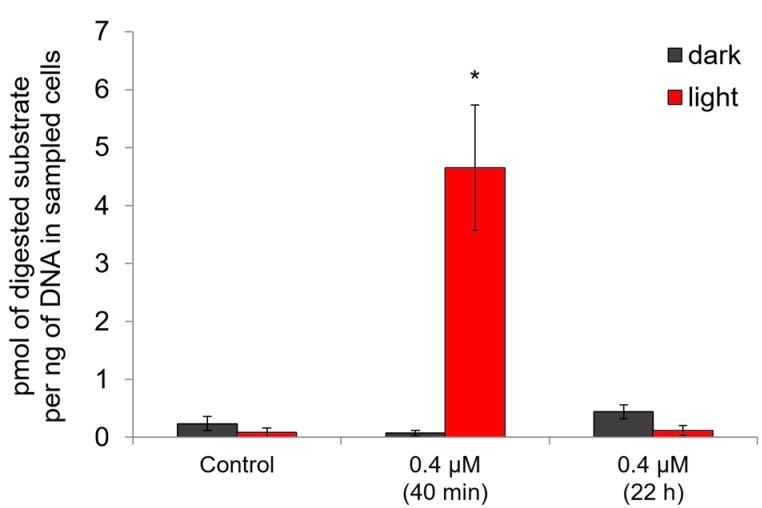
Caspase-3 activity in HeLa cells after treatment with Compound **21** in the dark (40 min and 22 h) and with photoactivation (2 h in the dark followed by 20 min under red light and then again 40 min or 22 h in the dark). The data were normalized according to the DNA content in the corresponding samples. The average values of the six experimental repeats are shown in the figure. *—the difference between the effect in the dark and after photoacivation is significant at *p* < 0.005.

It is likely that this type of distribution, in which we see a lot of highly damaged nucleotides forming a separate pool, is due to an increase in apoptosis rate when nucleases digest the DNA [15,16]. The results of caspase-3 activity analysis also indicate that apoptosis is one of the mechanisms of cell death in this case (Figure 7). Forty minutes after an exposure of HeLa cells for 20 min to red light in the presence of Compound **21** in a concentration of 0.4 μM, we registered an increase in caspase-3 activity compared to the control (*p* < 0.005), which means that apoptosis was induced. After 24 h caspase-3 activity decreases to the control level. Importantly, Photolon^TM^ drug used in this study for comparison did not induce caspase-dependent apoptosis [6].

In the Figure 5C, we can see that the nucleus is less stained by Compound **21** than the cytoplasm. This suggests that it either did not penetrate the nucleus, or penetrated but to a lesser degree than in the cytoplasm. In this case, such a rapid activation of caspase 3 and DNA fragmentation can be explained by direct damaging and permeabilization of mitochondrial membrane. Some compounds are known to permeabilize the mitochondrial outer membrane without activation of upstream signaling [17,18]. At the same time, we can not exclude the ability of Compound **21** to directly induce DNA damage given the fact of increasing the number of slightly fragmented nucleoids (Figure 6B) and activation of proapoptotic gene BAX after the photoinduced treatment (Table 3).

### 2.6. The Ability of Compound ***21*** to Destroy Anuclear Cells and to Oxidize Biomolecules

**Figure 8:**
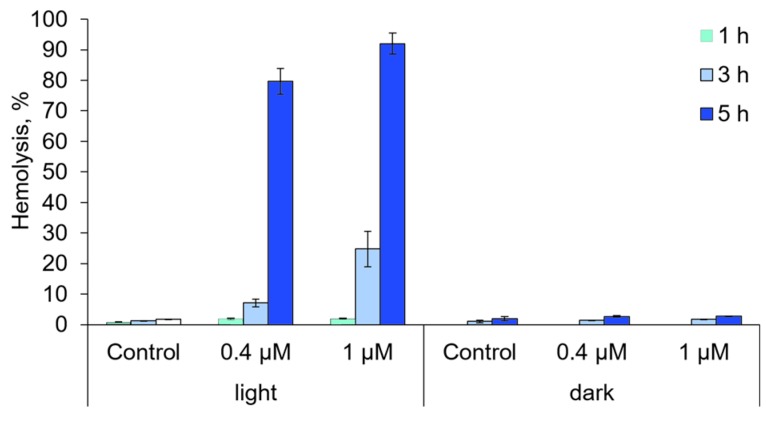
The degree of erythrocytes’ hemolysis during incubation with the tested Compound **21** (0.4 and 1 μM) for 1, 3, and 5 h.

**Figure 9:**
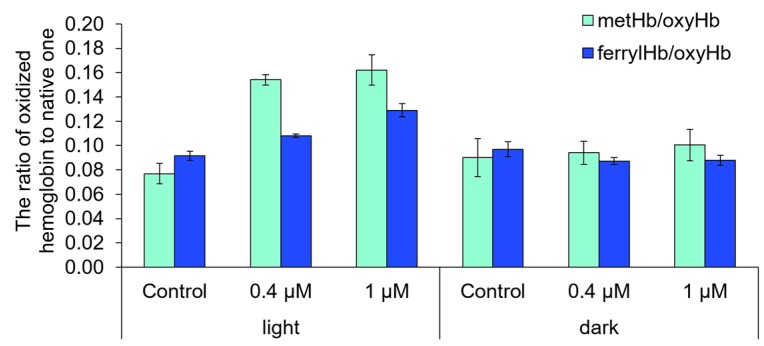
The relation of oxidized forms of hemoglobin (metHb and ferrylHb) to the native one (oxyHb) in erythrocytes’ hemolysates after 5 h of incubation with the tested Compound **21** (0.4 and 1 μM).

To measure the activity of Compound **21** on the protein and membrane structures in a nuclear-free system, we performed a phototoxicity test (Photo-RBC test) which combines the analysis of photohemolysis and hemoglobin oxidation [19,20]. The results of the test showed that the compound in concentrations of 0.4 and 1 μM does not induces hemolysis in the dark (Figure 8). At the same time was also indicated a significant increase in oxidized hemoglobin relative content in the erythrocytes’ hemolysates after incubation with the photoactivated compound. This means that Compound **21** is able to cause a photoinduced oxidation of biomolecules (Figure 9). Indirectly, the oxidizing activity of photoactivated Compound **21** additionally evidenced a tendency to increase the level of *GSR*, *SOD2*, and *PBP74* gene expression in HeLa cells.

## 3. Materials and Methods

### 3.1. Cell Culture

In the research, we used four human cell lines: cervical cancer (HeLa), pulmonary adenocarcinoma (A549), immortalized embryonic kidney epithelial cells (Hek293), and normal embryonic lung fibroblasts (HELF-104). The cells were cultured in a growth medium DMEM/F12 (PAA Laboratories GmbH, Cölbe, Austria) containing 10% of fetal bovine serum (FBS) (HyClone, Logan, UT, USA), without antibiotics at 37 °C and 5% CO_2_.

### 3.2. Estimation of Dark Cytotoxic Activity

Stock solutions of the analyzed compounds were prepared by dissolving in dimethylsulfoxide (DMSO) (Amresco, Solon, OH, USA) in different concentrations. One microliter of the stock solution of the tested compound in the corresponding concentration was added to 199 µL of the medium, containing 5000 HeLa cells in sterile culture plates. The final concentrations of the compounds varied from 0.01 up to 100 µM while DMSO was always 0.5% (*v*/*v*). The same concentration of DMSO was added to the reference samples. Cells with the studied compounds were cultured for 72 h at 37 °C, 100% humidity, and 5% CO_2_, in the dark. The amount of living cells was analyzed using a fluorimetric method according to Lindhagen and coauthors (FMCA method) [21]. To do this, a monolayer culture was washed with 200 µL of phosphate buffered saline (PBS) just after the medium had been removed. Then 100 µL of fluorescein diacetate (Sigma-Aldrich, St. Louis, MO, USA) solution in FDA-buffer was added to each well for 40 min incubation at 37 °C/5% CO_2_. All the manipulations were done under low light condition to avoid a photoinduced effect. Fluorescence of the incubated solution was measured at excitation wavelength 485 nm and emission 520 nm (Fluorat-02 Panorama, Lumex, St. Petersburg, Russia).

The experiments were performed in 9–12 independent microcultures for each concentration. Statistical analysis was carried with Statistica 6.0 software (StatSoft Inc., Tulsa, OK, USA) using Student’s *t*-criterion. The data was checked for artifacts using Grabbs criterion.

### 3.3. Estimation of Photoinduced Cytotoxic Activity

The tested compounds were added to the cell cultures as described above. The cells with the tested compounds were incubated for 2 h at 37 °C, 100% humidity, and 5% CO_2_. Then the cells were exposed to light (Light-emitting diodes, λ = 660 nm, the amount of light exposition 12 J/cm^2^) for 20 min. Later, the cells were put back into the CO_2_-incubator for 70 h. Then the number of living cells was determined using FMCA method as described above. All compounds were tested using HeLa cells (5000 cells/well). Estimation of photoinduced cytotoxic activity of Compound **21** was additionally performed using the following cell lines: HELF-104 (1000 cells/well), A549 (5000 cells/well), and HEK293 (5000 cells/well). Photosensitizing properties of Compound **21** were further tested using an exposure to λ = 700 nm light (Light-emitting diodes, the amount of light exposition 3.15 J/cm^2^) for 20 min. As a positive control a famous photosensitizing drug, Photolon^TM^ (RUE “Belmedpreparaty”, Minsk, Belarus), was used.

The experiments were performed in 9–12 independent microcultures for each concentration. Statistical analysis was carried as for dark cytotoxicity.

### 3.4. Analysis of DNA Damage with Comet-Assay

The level of DNA damage was estimated using the alkaline version of Comet-assay. HeLa cells (20,000 cells per well) were incubated with Compound **21** (0.4 and 1 μM) for 2 h in sterile culture 96-well plates at 37 °C and 5% CO_2_. Then the cells were exposed to light (λ = 660 nm) for 20 min. Later, the cells were put back into the CO_2_-incubator for 6 h. The cells with pure DMSO (0.5% (*v*/*v*)) and the cell with the compound without light exposure were used as two controls. After incubation, the medium was removed, cells were washed with 200 µL of PBS and detached with 20 µL of trypsin-EDTA solution. After trypsinization, 80 µL of medium was added and the resulting 100 µL of suspension was quickly mixed with 233 µL of 1% low melting point agarose prepared in PBS. One hundred fifty microliters of obtained mixture was immediately placed on slides, (pre-coated with 1% normal melting point agarose) and covered with cover-slips. After a 5 min cooling at 4 °C, cover-slips were gently removed and slides were immersed into a lysis buffer (2.5 M NaCl, 100 mM Na_2_EDTA, 10 mM Tris-HCl, pH 10.0, 10% DMSO, 1% Triton X-100) and incubated at 4 °C overnight. After lysis, the slides were placed into an alkaline solution (300 µM NaOH, 1 µM EDTA; pH = 13.0) for DNA unwinding (40 min, 4 °C). Electrophoresis was done in the same alkaline solution for 25 min at 1 V/cm. Then the slides were washed for 15 min in neutralizing solution (0.4 µM Tris, 10 µM HCl; pH = 7.5) and twice in distilled water for 7 min. Washed slides were placed in 95% ethanol for 10 min and dried. The processed slides were then stained with 100 µL of ethidium bromide (MERCK, Kenilworth, NJ, USA) (2 µg/mL) and covered with a cover glass. Images were captured using a fluorescent microscope Axioscope A1 (Carl Zeiss, Oberkochen, Germany) with CCD camera AxioCam ICm 1 and an AxioVision software (Carl Zeiss, Oberkochen, Germany). DNA damage level was assessed as Olive moment using CometScorePro software (TriTekCorp, Sumerduck, VA, USA) in a semi-automatic mode. From 50 to 80 cells per slide were analyzed. Six slides obtained with separately treated cultures were made for each data point. Mean values of the median Olive moments were calculated for each slide. Student’s *t*-criterion was used to estimate the statistical difference between the treatment variants.

### 3.5. Analysis of Caspase 3 Activity

Caspase 3 Assay Kit Fluorimetric (Sigma-Aldrich) was used to assess the induction of apoptosis. HeLa cells (15,000 cells per well) were incubated for 2 h with Compound **21** (0.4 µM) in culture 96-well plates at 37 °C and 5% CO_2_. Then exposed to red light (λ = 660 nm, 20 min) and incubated for 40 min and 21 h 40 min. Analogous conditions but without exposure to light were used in the control. After incubation, the medium was removed, cells were washed with 200 µL of PBS, and 25 µL of lysis solution from Caspase 3 Assay Kit Fluorometric (Sigma-Aldrich) was added. All subsequent procedures were performed according to the manufacturer’s protocol. Fluorescence was measured on “Fluorat-02 Panorama” (Lumex). The difference between the values obtained for the wells with and without specific Caspase 3 inhibitor was calculated to estimate a degree of the specific substrate digestion. Data were normalized according to the DNA concentrations in the corresponding lysates measured with PicoGreen^®^ (Molecular Probes, Eugene, OR, USA). The results were presented in pmol of the digested labeled substrate per amount of sample containing 1 ng of DNA based on the calibration ladder built using the clear fluorescent label, 7-amido-4-methylcoumarin.

### 3.6. Estimation of the Ability of Compound ***21*** to Penetrate the Plasmalemma

The ability of Compound **21** to penetrate into the cell was estimated using fluorescent microscopy. HeLa cells (5000 per well) were grown on a microscope slide with a 12-well silicone chamber (Ibidi GmbH, Planegg, Germany) in 199 µL of DMEM/F12 medium containing 10% (*v*/*v*) of fetal bovine serum. The cells were incubated with 1 µL of Compound **21** solution in DMSO (final concentration of compound was 1 µM) for 40 min at 37 °C, 100% humidity, and 5% CO_2_. After incubation, the medium with the studied compound and silicone chamber all were removed, cells were washed with PBS and covered with a cover glass. Images were captured under the transmitted light and fluorescent microscopy excitation wave 352–377 nm and emission >397 nm.

### 3.7. Analysis of Stress-Response Genes Expression

HeLa cells (40,000 per well) were incubated for 2 h with Compound **21** (0.4 µM) in a 96-well sterile culture plates at 37 °C and 5% CO_2_ then they were subjected to light (λ = 660 nm) for 20 min and continued to incubate for additional 3 h 40 min. Experiments were made in three parallel biological replications and three qPCR reactions within each replication. Each replication was carried out using combined sample from 10 wells. Cell suspension with pure DMSO (0.5% (*v*/*v*)) was used as the reference sample. After the incubation medium was removed, cells were washed with 200 µL of PBS, and 35 µL/well of lysis solution from Aurum Total RNA MiniKit (Bio-Rad, Hercules, CA, USA) was added. Then RNA was extracted from combined cell lysate from 10 wells per sample according to the manufacturer’s protocol. The quantity and quality of the extracted total RNA was estimated with the Experion automated electrophoresis system (Bio-Rad). Reverse transcription was made with Maxima First Strand cDNA Synthesis Kit (Thermo Scientific, Rockford, IL, USA) according to the manufacturer’s protocol. Real-time PCR was carried out using CFX96 PCR Detection System (Bio-Rad). Reaction mixture (20 µL) contained 20 ng of cDNA, primers (300 nM), and Maxima SYBR Green qPCR Master Mix (Thermo Scientific). The following PCR cycling conditions were used: 95 °C for 10 min, 40 cycles of 95 °C for 15 s, 60 °C for 60 s. Relative expression was calculated using the Δ*C*_t_ method by normalizing to the housekeeping genes *ACTB* and *GAPDH*. Sequences of primers (Table 4) were taken from articles or developed with Primer BLAST online service.

Primer specificity was validated by examining of PCR product melting curves using High Resolution Melt analysis mode of CFX96 PCR Detection System (Bio-Rad). Data were analyzed using CFX Manager (Bio-Rad), the following statistical manipulations were done using Statistica 6.0 (StatSoft Inc.) and Excel (Microsoft). Data were checked for artifacts by Grabbs criterion. Statistical significance of the effects was estimated with one-way ANOVA with *post*-*hoc* Neuman-Keuls test.

### 3.8. Hemolytic Activity Analysis

Dark cytotoxicity and photodynamic activity of Compound **21** in relation to anuclear cells was estimated according to their ability to induce hemolysis of erythrocytes in the dark and under the condition of continuous illumination (λ = 660 nm). In the experiments, 0.5% erythrocyte suspension (in PBS, pH 7.4) from the blood of outbred mice was used. DMSO solutions of Compound **21** were put into the erythrocyte suspension and incubated at 37 °C while being constantly slowly mixed in a thermostatic shaker ES-20 (Biosan, Riga, Latvia). Control samples had the corresponding amount of DMSO. The degree of hemolysis was estimated based on hemoglobin yield into the incubation media using the spectrophotometer, Genesys 20 (Thermo Scientific, Rockford, IL, USA) at λ = 524 nm. Hemolysis percentage was calculated based on the relation of the test sample hemolysis to the sample with full hemolysis [29]. Besides the degree of hemolysis, we also tested the ratio of different hemoglobin forms (oxyHb, metHb, and ferrylHb) [19]. For this purpose, we analyzed the hemolysate absorbance spectrum in the interval 540–630 nm using the spectrofluorimeter “Fluorate-02-Panorama” (Lumex). The content of different hemoglobin forms (oxyHb, metHb, and ferrylHb) was calculated based on the corresponding molar absorbance coefficients [30].

## 4. Conclusions

The dark and photoinduced cytotoxic activity of 24 new chlorophyll-a derivatives which contain the substituents of oligoethylene glycol on the periphery of their macrocycles was estimated.

It is shown that all the new compounds have high photoinduced cytotoxicity. At the same time, pyropheophorbide-a 17-diethylene glycol ester (Compound **21**) showed the higher photoinduced cytotoxic activity and one of the lower dark cytotoxicities. Using the fluorescent microscopy, we determined that Compound **21** is able to quickly penetrate into the cell. In the activated state Compound **21** also causes caspase-dependent apoptosis. Using Comet assay was demonstrated the high degree of the DNA fragmentation in treated cells, which can be the result of apoptotic DNA degradation and possible direct damage by light-activated Compound **21**. An increase in expression of cell cycle control genes (*p21*, *p16*) demonstrates an activation of signal cascades which leads to the cell cycle arrest. The experiments with nuclear-free mice erythrocytes showed the ability of this compound to cause photoinduced oxidation of biomolecules. In addition, it was shown that Compound **21** is effectively activated with low-energy 700 nm light, which can penetrate deep into the tissue. Mentioned properties make Compound **21** a promising substance for development of the new drugs for photodynamic therapy of cancer.

## Figures and Tables

**Table 1:** Dark cytotoxic activity of chlorophyll-a derivatives and oligoethylene glycols on HeLa cells.

Compound	IC_50_ ± SE, μM	Compound	IC_50_ ± SE, μM	Compound	IC_50_ ± SE, μM
**1**	57.51 ± 38.21	**11**	1.43 ± 0.21	**21**	>100
**2**	38.40 ± 23.26	**12**	1.94 ± 0.78	**22**	54.70 ± 27.13
**3**	48.36 ± 36.18	**13**	1.67 ± 0.34	**23**	12.77 ± 1.20
**4**	21.32 ± 3.67	**14**	16.64 ± 5.13	**24**	5.58 ± 0.93
**5**	22.61 ± 3.73	**15**	13.15 ± 2.08	**25**	6.88 ± 5.06
**6**	19.72 ± 2.79	**16**	3.50 ± 0.84	Diethylene glycol	>100
**7**	13.88 ± 2.92	**17**	12.09 ± 1.85	Triethylene glycol	>100
**8**	19.09 ± 6.00	**18**	6.47 ± 1.33	Tetraethylene glycol	>100
**9**	24.93 ± 5.73	**19**	41.56 ± 27.36	Pentaethylene glycol	>100
**10**	34.28 ± 11.39	**20**	1.97 ± 0.88	Hexaethylene glycol	>100
				Photolon^TM^	31.09 ± 4.92

IC_50_—concentration at which cell grown was inhibited by 50% after 72 h incubation with the compound. Calculated from the equation of compound concentration in relation to the survival index; SE—Standard Error.

**Table 2:** The values IC_50_ ± SE (μM) for photoinduced cytotoxicity of Compound **21** and the drug Photolon^TM^ in the experiments in vitro.

Compound	λ, nm	HeLa	A549	Hek293	HELF-104
**21**	660 nm	0.15 ± 0.03	0.07 ± 0.01	0.48 ± 0.23	0.16 ± 0.06
	700 nm	0.23 ± 0.02	0.21 ± 0.03	>1	0.39 ± 0.12
Photolon^TM^	660 nm	>1	>1	0.44 ± 0.09	>1

IC_50_—concentration at which cell grown was inhibited by 50% after treatment included 2 h preincubation, photoactivation for 20 min, and subsequent 70-h incubation in the dark. The values were calculated from the equation which describes the relationship between the survival index and the compound concentration; SE—Standard Error.

**Table 3:** Relative expression of genes of stress-response systems in HeLa cells after treatment with Compound **21** (0.4 μM) in the dark and with photoactivation.

Gene	Dark Toxicity		Phototoxicity	
	Control	0.4 µM	Control	0.4 µM
*CDKN1A*	0.363 ± 0.093	0.233 ± 0.024	0.357 ± 0.052	0.641 ± 0.083 **
*CDKN2A*	0.963 ± 0.119	1.017 ± 0.108	0.713 ± 0.100	0.869 ± 0.137
*CDKN2D*	0.964 ± 0.062	0.992 ± 0.057	0.908 ± 0.032	1.248 ± 0.076 **
*BAX*	1.064 ± 0.044	1.038 ± 0.018	0.992 ± 0.033	1.289 ± 0.035 **
*TNFSF10*	0.856 ± 0.103	0.781 ± 0.055	0.656 ± 0.078	0.768 ± 0.067
*GSR*	0.935 ± 0.126	1.079 ± 0.063	1.216 ± 0.042	1.127 ± 0.068
*PBP74*	0.424 ± 0.024	0.449 ± 0.027	0.455 ± 0.010	0.544 ± 0.025 *
*SOD2*	1.002 ± 0.127	1.112 ± 0.079	1.196 ± 0.049	1.428 ± 0.169

The table demonstrates the mean values of nine reactions (3 biological repeats × 3 technical repeats) and ±SEM. * and **—the difference between dark toxicity and phototoxicity is significant at *p* < 0.05 and *p* < 0.01 (ANOVA with post-hoc Newman-Keuls test).

**Table 4:** Primer sequences for gene expression analysis.

Gene	Forward Primer	Reverse Primer	Source
*BAX*	AGAGGATGATTGCCGCCGT	CAACCACCCTGGTCTTGGAT	[22]
*TNFSF10*	GCTGAAGCAGATGCAGGACAAG	CTGACGGAGTTGCCACTTGAC	[23]
*CDKN1A*	CAGCAGAGGAAGACCATGTG	GGCGTTTGGAGTGGTAGAAA	[24]
*CDKN2A*	GACATCCCCGATTGAAAGAA	TTTACGGTAGTGGGGGAAGG	*
*CDKN2D*	TCAGTTTCTTCTGCGCCTCA	CAACGTGCACACTTCAGGTC	*
*SOD2*	GCTGACGGCTGCATCTGTT	CCTGATTTGGACAAGCAGCAA	[25]
*GSR*	ATCCCCGGTGCCAGCTTAGG	AGCAATGTAACCTGCACCAACAA	[26]
*PBP74*	TCTGGACTGAATGTGCTTCG	ATCCCCATTTGTGGATTTCA	[27]
*GAPDH*	ACACCCACTCCTCCACCTTTG	GCTGTAGCCAAATTCGTTGTCATAC	[23]
*ACTB*	GCGCGGCTACAGCTTCA	CTTAATGTCACGCACGATTTCC	[28]

* Developed using Primer BLAST online service: http://www.ncbi.nlm.nih.gov/tools/primer-blast/.

## Abbreviations

FMCA	Fluorometric microculture cytotoxicity assay
IC_50_	Concentration of compond at which cell grown was inhibited by 50%
DMSO	Dimethyl sulfoxide
Photo-RBC test	Photo-Red Blood Cells test
oxyHb	oxyhemoglobin
metHb	methemoglobin
ferrylHb	ferrylhemoglobin
